# *Brucella* Genetic Variability in Wildlife Marine Mammals Populations Relates to Host Preference and Ocean Distribution

**DOI:** 10.1093/gbe/evx137

**Published:** 2017-07-20

**Authors:** Marcela Suárez-Esquivel, Kate S. Baker, Nazareth Ruiz-Villalobos, Gabriela Hernández-Mora, Elías Barquero-Calvo, Rocío González-Barrientos, Amanda Castillo-Zeledón, César Jiménez-Rojas, Carlos Chacón-Díaz, Axel Cloeckaert, Esteban Chaves-Olarte, Nicholas R. Thomson, Edgardo Moreno, Caterina Guzmán-Verri

**Affiliations:** 1 Programa de Investigación en Enfermedades Tropicales, Escuela de Medicina Veterinaria, Universidad Nacional, Heredia, Costa Rica; 2 Pathogen Genomics, Wellcome Trust Sanger Institute, Hinxton, United Kingdom; 3 Institute for Integrative Biology, University of Liverpool, United Kingdom; 4 Servicio Nacional de Salud Animal, Ministerio de Agricultura y Ganadería, Heredia, Costa Rica; 5 Centro de Investigación en Enfermedades Tropicales, Facultad de Microbiología, Universidad de Costa Rica, San José, Costa Rica; 6 ISP, INRA, Université François Rabelais de Tours, UMR 1282, Nouzilly, France

**Keywords:** *Brucella*, marine mammals, genome degradation

## Abstract

Intracellular bacterial pathogens probably arose when their ancestor adapted from a free-living environment to an intracellular one, leading to clonal bacteria with smaller genomes and less sources of genetic plasticity. Still, this plasticity is needed to respond to the challenges posed by the host. Members of the *Brucella* genus are facultative-extracellular intracellular bacteria responsible for causing brucellosis in a variety of mammals. The various species keep different host preferences, virulence, and zoonotic potential despite having 97–99% similarity at genome level. Here, we describe elements of genetic variation in *Brucella ceti* isolated from wildlife dolphins inhabiting the Pacific Ocean and the Mediterranean Sea. Comparison with isolates obtained from marine mammals from the Atlantic Ocean and the broader *Brucella* genus showed distinctive traits according to oceanic distribution and preferred host. Marine mammal isolates display genetic variability, represented by an important number of IS*711* elements as well as specific IS*711* and SNPs genomic distribution clustering patterns. Extensive pseudogenization was found among isolates from marine mammals as compared with terrestrial ones, causing degradation in pathways related to energy, transport of metabolites, and regulation/transcription. *Brucella ceti* isolates infecting particularly dolphin hosts, showed further degradation of metabolite transport pathways as well as pathways related to cell wall/membrane/envelope biogenesis and motility. Thus, gene loss through pseudogenization is a source of genetic variation in *Brucella*, which in turn, relates to adaptation to different hosts. This is relevant to understand the natural history of bacterial diseases, their zoonotic potential, and the impact of human interventions such as domestication.

## Introduction

Bacteria living in isolation or stable habitats, such as the intracellular milieu, tend to have clonal populations with smaller and degraded genomes than free-living ancestors, which keep larger and more versatile genomes (Moreno 1998; Toft and Andersson 2010). Still, some versatility must be preserved in order to confront environmental challenges.

Most of the emergent human pathogens have a zoonotic origin where transgression of host barriers is critical (Greger 2007; Jones et al. 2008). Understanding how microorganisms are able to surpass such barriers, particularly host range adaptation is relevant to comprehend the emergence of pathogens.

It has been proposed that genetic drift and speciation in extant clonal bacteria will depend exclusively on mutation and internal genetic rearrangements (Moreno 1997). Several mechanisms had been described in mammal bacterial pathogens with small genomes to keep genetic variability (Bolotin and Hershberg 2015). However, it is possible that these mechanisms are underrepresented when studying bacterial pathogens of domesticated animals. In this sense, domestication may represent a microbial population bottleneck for diversity: By selecting animals genetically suited for human benefit, there is probably selection of their microorganisms. Within this context, to study bacteria infecting wildlife populations, closely related to bacteria isolated from domesticated animals, may bring light to pathways followed by these selection processes.

Members of the *Brucella* genus are facultative extracellular intracellular α 2-Proteobacteria responsible for causing brucellosis in a variety of mammals. This chronic disease results in abortion and infertility in livestock causing economic losses mainly in middle and low income countries (Moreno and Moriyón 2006). Humans are infected through contaminated animal-derived food products or infected animals. It is considered by the WHO as a “forgotten neglected zoonosis”, estimating that for every reported human case, there are 25–50 unreported cases (World Health Organization 2014).

*Brucella* species share 97–99% identity at genome level. In spite of this close genetic relatedness and genomes with no lysogenic phages or detected plasmids, there is a strong correlation between genotypes, virulence, and host preference (Moreno and Moriyón 2006). These traits make *Brucella* an appropriate model for understanding bacterial host adaptation. Interestingly, pseudogene accumulation in prokaryotes has been demonstrated as a hallmark of recent host adaptation. It is also inversely related to host-range, that is, narrow host-range pathogens tend to have a higher number of pseudogenes, and similar phenomena had been studied in *Brucella* (Chain et al. 2005; Tsolis et al. 2009; Wattam et al. 2009; Goodhead and Darby 2015).

Here we used *Brucella* isolates from free-living marine mammals in three of the world's major oceanic basins to look for elements of genetic variation and their relation to host specialization of this zoonotic pathogen. We characterized *Brucella ceti* isolates from dolphins from the Pacific Ocean and the Mediterranean Sea, and compared them with isolates obtained from marine mammals (dolphins, porpoises, and seals) from the Atlantic Ocean. The distinctive traits observed among the isolates showed signatures of host preference, speciation, and oceanic distribution. Expanding that comparison to *Brucella* sp. isolates, revealed genetic variability elements among isolates from wildlife marine mammals as compared with those from terrestrial domesticated animals. This variability is demonstrated through a SNPs and IS*711* specific clustering pattern across genomes and a higher number of IS*711* elements. There is also an important number of pseudogenes affecting specific metabolic pathways and inducing gene loss according to host preference. Therefore, gene loss should be considered a source of genetic variation in *Brucella*, which in turn, relates to adaptation to different niches and host preference.

## Materials and Methods

### Bacterial Strains

The list of isolates used in this study is presented in supplementary data set S1, Supplementary Material online and includes 23 *B. ceti* isolates from stranded striped dolphins from the Eastern Tropical Pacific of Costa Rica as well as several previously described isolates: Four from the Mediterranean Sea, nine from the North Atlantic Ocean, one from France, four *Brucella pinnipedialis* from the North Atlantic Ocean, and one *Brucella* sp. from California. These were analyzed alongside with reference strains from other *Brucella* species (*Brucella abortus*, *Brucella canis*, *Brucella melitensis*, *Brucella microti*, *Brucella neotomae*, *Brucella ovis* and *Brucella suis*).

### *Brucella* Phenotypic Characterization

All procedures involving live *Brucella* were carried out according to the “Reglamento de Bioseguridad de la CCSS 39975-0”, year 2012, after the “Decreto Ejecutivo #30965-S”, year 2002 and research protocol NFEG06 approved by the National University, Costa Rica. Phenotypic analysis of *Brucella* isolates was carried out as described (Hernández-Mora et al. 2008). Matrix-assisted laser desorption/ionization time-of-flight mass spectrometry (MALDI-TOF MS) studies of *Brucella* protein extracts and gas chromatographic analysis of fatty acid methyl esters were performed as previously described (Isidoro-Ayza et al. 2014). A dendogram derived from the analysis of concatenated data based on the retention time of the fatty acid methyl esters, and on the protein masses detected, was constructed using an Agglomerative Hierarchical Clustering (AHC) algorithm, using Microsoft Excel 2000/XLSTAT-Pro (Version 4.07, 2013, Addinsoft, Inc., Brooklyn, NY). Proximities were calculated using Squared Euclidean Distance, and aggregation was calculated using the unweighted pair-group average method. Raw data are in supplementary data set S2, Supplementary Material online.

### DNA Molecular Studies

DNA was extracted with DNeasy Blood & Tissue kit from QIAGEN or Promega Wizard Genomic DNA Purification kit, and stored at −70 °C until used.

Multiple loci variable number of tandem repeats (MLVA-16) analysis and the corresponding cladograms were generated according to described protocols (Le Flèche et al. 2006; Al Dahouk et al. 2007; Maquart et al. 2009; Isidoro-Ayza et al. 2014) using the MLVA-NET database (http://microbesgenotyping.i2bc.paris-saclay.fr/ (last accessed July 24, 2017); Grissa et al. 2008). Values obtained for each MLVA marker are in supplementary data set S2, Supplementary Material online. DNA polymorphism at the *omp2* locus was performed as described (Cloeckaert et al. 2001).

Other genotyping techniques such as multiplex PCR Bruce-ladder, MLST, PCR detection of ST27 or *bcsp31*, HRM RT-PCR and PCR targeting specific IS*711* elements, were performed either as previously described (references in data set S1, Supplementary Material online) or in silico (supplementary data set S3, Supplementary Material online).

Whole genome sequencing (WGS) was performed at the Wellcome Trust Sanger Institute on Illumina platforms according to in house protocols (Quail et al. 2009, 2012). For WGS assembly and alignment sequencing reads were de novo assembled using Velvet Optimiser (Zerbino and Birney 2008) and contigs were ordered using abacas (Assefa et al. 2009) against *B. abortus* 9-941 under accession numbers NC_006932 and NC_006933 at the National Center for Biotechnology Information (NCBI). To detect miss-assemblies, raw data were mapped back against the genome assemblies using SMALT v.0.5.8 (http://www.sanger.ac.uk/science/tools/smalt-0; last accessed July 24, 2017). All sequencing data have been deposited at the European Nucleotide Archive (ENA) (http://www.ebi.ac.uk/ena/; last accessed July 24, 2017) under the accession codes listed in Supplementary data set S1, Supplementary Material online. Other WGS sequences from various *Brucella* strains used for comparative purposes were obtained from GenBank (supplementary data set S1, Supplementary Material online). Incomplete genomes, or low N50 scaffolds from databases were not included in the analysis.

### Phylogenetic Reconstruction

To construct a multiple sequence alignment for phylogenetic reconstruction, whole-genome sequence data from two *Ochrobactrum* species and the *Brucella* isolates from different hosts (Supplementary data set S1, Supplementary Material online) were aligned by bwa and mapped with SMALT v.0.5.8 against *B. abortus* 9-941, with an average coverage of 98.81%. Single Nucleotide Polymorphisms (SNPs) were called using samtools (Li et al. 2009), and 311,780 variable sites were extracted using snp sites (Page et al. 2016). The resulting alignment was used for maximum likelihood phylogenetic reconstruction with RAxML v7.0.4 (Stamatakis 2006). The phylogenetic tree was rooted using *Ochrobactrum anthropi* ATCC49188 and *Ochrobactrum intermedium* strain type LMG3301. Within this data set the *B. ovis* lineage shared the most recent common ancestor with *Ochrobactrum*, therefore it was subsequently used to root phylogenies constructed using only *Brucella*.

All analyses relevant to reference annotation (e.g., dN/dScalculation and SNP ascription to coding sequences—CDS) were relative to *B. abortus* 9-941 (accession numbers NC_006932 and NC_006933). The alignment and the tree files were used to generate a tab file containing coordinates of SNPs position relative to the root; all three files were used to produce a visual reconstruction of the SNPs distribution along the genome per branch (as seen in supplementary fig. S5, Supplementary Material online).

### Comparative Genomics from Whole Genome Sequences

Comparative genomics was facilitated by annotation of *B. ceti* draft genome assemblies by Prokka (Seemann 2014) and by annotation transfer from *B. abortus* 2308 Wisconsin (Suárez-Esquivel et al. 2016). The annotation of genes absent in *B. abortus* 2308 Wisconsin was completed through manual comparison against reference genomes (Supplementary data set S1, Supplementary Material online): *B. suis* 1330, *B. ovis* ATCC 25840, *B. melitensis* 16M and *B. pinnipedialis* B2/94. We identified orthologous protein groups and the number of new, conserved and total genes added by each genome included in the analysis (discovery rate) by using Roary (Page et al. 2015).Visualizations were done with Artemis and comparisons with the Artemis Comparison Tool (ACT; Carver et al. 2005). The presence of recombination events was analyzed by Genealogies Unbiased By recomBinations In Nucleotide Sequences (Gubbins) (Croucher et al. 2014).

#### Pseudogene Analysis

To detect pseudogenes in *B. ceti*, we selected five phylogenetically representative draft genomes from marine mammal *brucellae* (*B. ceti* bmarCR17 -P1 cluster-, *B. ceti* bmarMR26 -MR cluster-, *B. ceti* M644/93/1 -A1 cluster-, *B. ceti* M187/00/1 -A2/B cluster-, and *B. pinnipedialis* M2466/93/4 -C2 cluster-) and automatically transferred the annotation of the manually curated draft genome working strain *B. abortus* 2308 Wisconsin (Suárez-Esquivel et al. 2016).

Pseudogenes were defined as any gene containing deletions or insertions that removed start or stop codons, or at least one in-frame stop codons and/or frame shifts compared with orthologs in *B. abortus* 2308 Wisconsin or reference genomes as described above. Pseudogenes were detected manually using Artemis and ACT. Pseudogenes from marine mammal brucellae with no homologs in terrestrial *Brucella* were compared against the NCBI nonredundant protein database using BlastX. The putative cellular localization was predicted by PSORT and the function was classified based on: The product description in the references annotation; BLAST comparison with several *Brucella* species and other genus; metabolic assigned pathway according to KEGG (Kanehisa et al. 2016). In depth metabolic pathway analysis of pseudogenes from particular phylogenetic branching points was carried out using BioCyc (Caspi et al. 2014).

#### Specific Search for Regions of Interest

In order to examine relevant phenotypic genes (virulence related, outer membrane, lipopolysaccharide [LPS] and flagellar genes), regions of interest were examined through bwa alignment and SMALT mapping. The number of SNPs, insertions and deletions in each one of the genes was recorded.

The number and position of the insertion sequence IS*711* were searched in the analyzed genomes by mapping the reads to the 842 bp IS*711* of *B. ovis* (accession number M94960). Those reads that showed 99% mapping, were then mapped against the reference WGS *B. ovis* ATCC 25480 in order to judge where IS*711* might be inserted. The reads that mapped >90% to the WGS were filtered to 50× coverage and used to produce a visual representation displaying the identified sites per genome and approximate location according to *B. ovis* sequence coordinates.

The presence, orientation, and distribution of 24 previously reported genomic islands (GIs) or anomalous regions (regions apparently acquired by horizontal gene transfer; Mancilla 2012; Rajashekara et al. 2004; Wattam et al. 2009) were examined across the four phylogenetically representative *B. ceti* genomes (see above). For this, a “genomic-island pseudo-molecule” was formed by concatenation of 23 genomic regions obtained from nonmarine *Brucella* reference sequences (supplementary data set S1, Supplementary Material online). Islands were concatenated and ordered as follows: GI-4, GI-3, SAR 1-2, wbk, SAR 1-5, GI-2, GI-1, SAR 1-17, 4 kb, 13 kb, GI-9, GI-8, 26.5 kb, IncP, 12 kb, GI-7, GI-6, GIBs2, GIBs3, SAR 2-10, GI-5, mtgC, and virB.

A BLAST comparison between the representative *B. ceti* genomes and the pseudo-molecule was performed and visualized using ACT. The described orientation of the islands was checked in several reference genomes (*B. suis* 1330, *B. microti* CCM 4915, *B. abortus* 9-941, and *B. ovis* ATCC 25840) to confirm the presence of inversions. The 24th GI, a 67 kb sequence found mainly in isolates from marine mammals (Audic et al. 2011; Maquart et al. 2008; Bourg et al. 2007) was similarly analyzed independently.

## Results and Discussion

### *Brucella ceti* Clusters According to Geographical Region and Host Type

To study host preference in nondomesticated *Brucella* hosts, we performed genotypic analysis of *B. ceti* isolated from dolphins from the Pacific Ocean and the Mediterranean Sea (table 1), and compared the results with those of isolates obtained from marine mammals (dolphins, porpoises, and seals) from the Atlantic Ocean. These findings were then related to host and geographical origin.

**Table 1:** Marine Mammal *Brucella* Isolates Used for WGS Analysis (detailed information in supplementary data set S1, Supplementary Material online)

Species/Host	N. of Isolates	Location^a^
***B. ceti***		
* Balaenoptera acutorostrata* (minke whale)	1	NA
* Delphinus delphis* (common dolphin)	2	NA
* Lagenorhynchus acutus* (Atlantic white-sided dolphin)	1	NA
* Phoca vitulina* (common seal)	1	NA
* Phocoena phocoena* (porpoise)	3	NA
* Stenella coeruleoalba* (striped dolphin)	27	ETP, MS, NA
* Tursiops truncatus* (bottle nose dolphin)	2	MS, France
***B. pinnipedialis***		
* Balaenoptera acutorostrat*a (minke whale)	1	NA
* Lutra lutra* (otter)	1	NA
* Phoca vitulina* (common seal)	2	NA
***Brucella* sp.**		
* Tursiops truncatus* (bottle nose dolphin)	1	USA

a NA, North Atlantic. ETP, Eastern Tropical Pacific. MS, Mediterranean Sea.

**Fig. 1.—:**
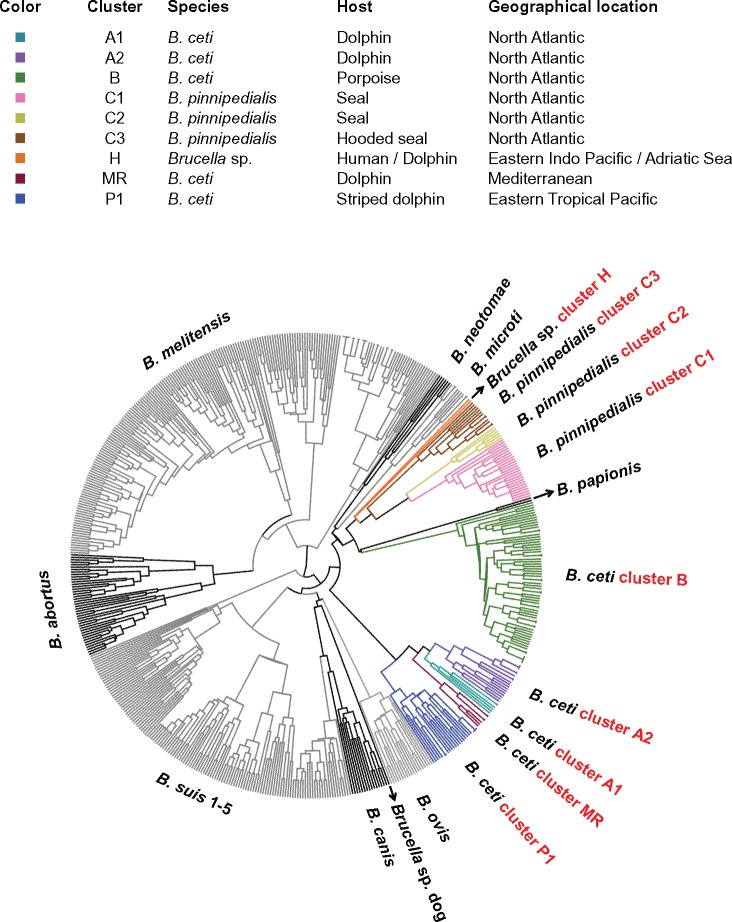
MLVA-16 analysis dendogram of *Brucella* related to geographic location and host. Analysis was performed according to: http://microbesgenotyping.i2bc.paris-saclay.fr/ (last accessed July 24, 2017). Increased resolution of marine isolates shown in supplementary fig. S1, Supplementary Material online.

MLVA-16 results were analyzed in the context of a worldwide *Brucella* databank and indicated that isolates from marine mammals showed dispersion and clustering according to the host from which they were isolated (fig. 1). Five *B. ceti* clusters were observed; two correspond to isolates mainly from different dolphin species (clusters A1 and A2) inhabiting the North Atlantic Sea. A third one is represented mostly by isolates from porpoises (cluster B) from the same sea (Maquart et al. 2009). Two additional *B. ceti* clusters affecting dolphins from the Pacific Ocean and the Mediterranean Sea were evident (Guzmán-Verri et al. 2012; Garofolo et al. 2014; Isidoro-Ayza et al. 2014). These clusters are herein referred as P1 and MR, respectively. The new isolates described in this study from the Eastern Tropical Pacific of Costa Rica belong to the P1 cluster affecting striped dolphins (*Stenella coeruleoalba*) (table 1, supplementary fig. S1, Supplementary Material online).

*Brucella pinnipedialis* isolated from the North Atlantic Sea was divided in three different MLVA-16 clusters that also related to host preference: Two were represented by isolates mainly from harbor seals (*Phoca vitulina*) (clusters C1 and C2) and one was represented by isolates from hooded seals (*Cystophora cristata*, cluster C3; Maquart et al. 2009). In addition, a human *Brucella* sp. isolate from New Zealand (*Brucella* sp. 02611), with no zoonotic link, an isolate from an aborted dolphin (*Brucella* sp. F5/99), and isolates from a stranded bottlenose dolphin from the Adriatic Sea (Cvetnić et al. 2016) define a distinct cluster (Maquart et al. 2009) herein named cluster H.

To determine if the dispersion and clustering observed by MLVA-16 could be reproduced by using higher resolution methods and establish possible explanations for this, we performed WGS of *Brucella* isolates from marine mammals from the North Atlantic, Eastern Tropical Pacific and Mediterranean Sea and analyzed them together with publically-available high quality *Brucella* genomes (table 1, supplementary data set S1, Supplementary Material online). The number of studied genomes (*n* = 50) was adequate to describe basic genomic characteristics of the genus, because the pan and core genome reached a plateau value within the data set (supplementary fig. S2, Supplementary Material online).

**Fig. 2.—:**
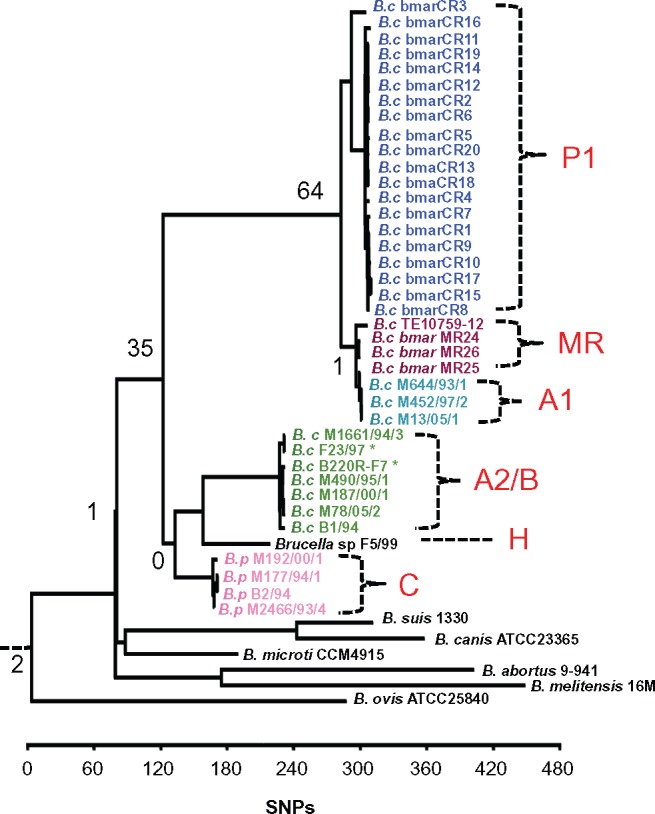
Whole genome sequence analysis of marine mammal *Brucella* shows phylogenetic correlation to host and geographic location. Phylogenetic tree based on 24,340 SNPs of different *Brucella* WGS. The isolates related to marine mammals showed six clusters, corresponding to those revealed by MLVA-16 analysis: P1, MR, A1, A2/B (which includes isolates from MLVA-16 A2—marked with asterisk—and B clusters), H and C. *Ochrobactrum* sp., used as the original root for the tree, was trimmed from the figure to increase tree resolution. Each cluster defining branch showed a 100 bootstrap value. The number of pseudogenes found is indicated in each defining node. Core genome analysis displayed similar tree topology.

The overall genetic structure of *Brucella* from marine mammals is in tune with the classical pathogenic *Brucella* from land mammals. Some conserved traits are: Presence of two chromosomes, absence of plasmids, no major recent recombination events, similar GIs/anomalous regions, and conservation of genes encoding virulence factors (fig. 2, supplementary figs. S3, S4, and supplementary data set S3, Supplementary Material online). Phylogenetic analysis using *O. anthropi* ATCC49188 and *O. intermedium* LMG3301 as an outgroup showed that *B. ovis* shared the most recent common ancestor within this data set with *Ochrobactrum*, so it was subsequently used to root phylogenies constructed using only *Brucella* isolates. When *Ochrobactrum* was excluded from the alignment, a total of 24,340 SNPs were found among the *Brucella* genomes. Of these, 19,081 SNPs were located in coding regions with a dN/dS ratio of 1.61.

The general topology of the SNPs based phylogenetic tree was consistent with those of similar studies using mainly terrestrial isolates, showing a clonal genus (Wattam et al. 2014, 2009) (fig. 2) or when *B. suis* 1330 was used as reference genome. It is also similar to a dendogram obtained by concatenation of results of matrix-assisted laser desorption/ionization time-of-flight mass spectrometry (MALDI-TOF MS) with gas liquid chromatography analysis of the fatty acid methyl esters (GLC) of *Brucella* cell extracts (supplementary fig. S5*A*, Supplementary Material online). When compared with the MLVA-16 study (fig. 1 and supplementary fig. S1, Supplementary Material online), the WGS analysis showed at least four *B. ceti* clusters, corresponding to MLVA-16 clusters P1, MR, and A1. The MLVA-16 clusters A2 and B are grouped in a single cluster that we refer as the A2/B genotype. The H cluster was represented by *Brucella* sp. F5/99, had its most recent common ancestor with *B. pinnipedialis* C cluster, and was also closely related to the *B. ceti* A2/B cluster.

When SNPs positions across each genome are visualized relative to the tree root, a barcode-like pattern due to different SNPs density regions within the genomes was observed. Some SNPs clusters could be identified, specific for a group of *Brucella* genotypes from marine mammals, or a single genotype (supplementary fig. S6, Supplementary Material online).

All together, these results expand the panorama observed in previous genotypic studies (Audic et al. 2011; Garofolo et al. 2014; Wattam et al. 2014; Maquart et al. 2009) and indicate a correlation between the evolutionary traits of *Brucella* isolated from marine mammals, its geographical origin and preferred host.

In order to benchmark other molecular techniques described for identification or typing of *Brucella*, we compared results generated using ten different techniques to the WGS classifications of the marine mammals *Brucella* isolates. Of these, multiplex PCR Bruce-ladder, adopted by the OIE for identification of *Brucella* species (OIE 2009) was able to classify but not discriminate all marine isolates (Supplementary data set S2, Supplementary Material online). Phylogenetic analysis based on DNA polymorphism at the *omp2* locus essentially replicated the genomic and phenotypic analysis results (supplementary fig. S5*B*, Supplementary Material online).

### Multiple Sources and Consequences of *B. ceti* Genome Variation

To establish if there were genetic traits that could be related to *Brucella* host preference and virulence using isolates from wild animals, a detailed analysis of the genome structure of *B. ceti* clusters as compared with other *Brucella* genomes was performed.

Analysis of amount of SNPs found in genes encoding virulence traits such as the type IV secretion system *virB*, some of its effectors (see below), LPS, membrane lipids, BvrR/BvrS two component system regulatory network and flagella did not show significant variations among the isolates (Supplementary data set S2, Supplementary Material online).

**Fig. 3.—:**
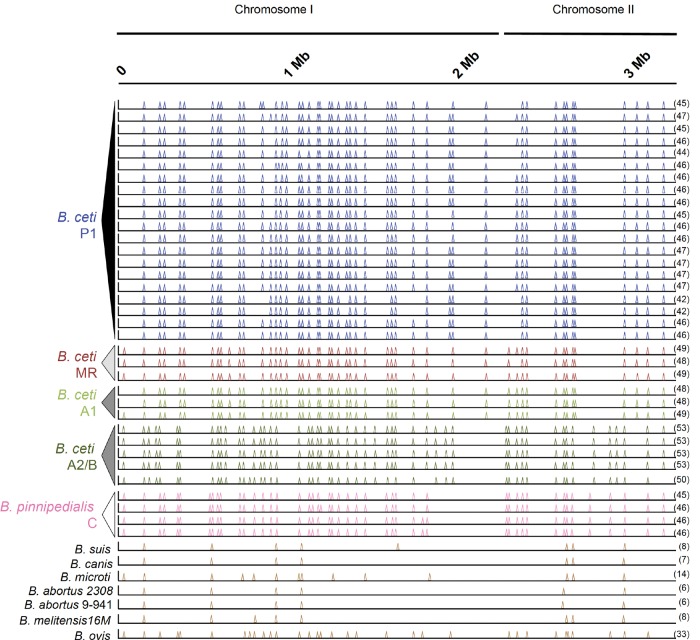
IS*711* insertion signatures for *Brucella* sp. Each peak represents the location of 50× coverage IS*711* insertion. The position in the first and second chromosomes (shown as a concatenated molecule) is indicated by the scale bar (in Mb) above. The number of IS*711* insertions is shown in parentheses at the end of each genome.

Genome alteration through the active transposon insertion sequence IS*711*, used as a *Brucella* genus fingerprint (Ocampo-Sosa and García-Lobo 2008), was examined. An increased number of this element was detected in *brucellae* from marine mammals as compared with those from terrestrial strains (fig. 3), consistent with previous reports (Bricker et al. 2000; Dawson et al. 2008; Bourg et al. 2007; Audic et al. 2011). This indicates that marine isolates show greater genome variability than terrestrial ones. Interestingly, several IS*711* insertion patterns along the genome assemblies were observed and related to phylogenetic position. Some variation among isolates within phylogenetic clusters was also observed (e.g., Cluster P1, fig. 3).

To study the *en bloc* gain or loss of syntenic genes across the *Brucella* isolates from marine mammals, further detailed comparative genomics of representative from each of the four genome clusters P1, MR, A1, and A2B was performed. Presence of previously reported 24 GIs, important as evidence of gene horizontal transfer and gain of virulence traits within the genus (Mancilla 2012) was investigated (supplementary fig. S4, Supplementary Material online). Inversion of a GI as compared with reference sequences was a frequent event found in all four genomes, particularly those found in chromosome II. The 12 kb and the 26.5 kb GIs were absent in all four genomes. GI-1 was absent in the P1 cluster and as previously reported, GI-3 was absent in the A2/B cluster representative (Wattam et al. 2014). The *wbk* GI, related to LPS synthesis, a virulence factor, has a particular rearrangement in the P1 cluster representative, caused by transposon and IS derived elements. However, they do not affect codifying genes as compared with the *B. melitensis* 16M *wbk* GI. The 67 kb GI related to *B. pinnipedialis* and to cluster H (Bourg et al. 2007; Audic et al. 2011) was found in the *B*. *pinnipedialis* isolates included in this study and in *B. ceti* bmarMR24. GI IncP was absent in *B. pinnipedialis* B2/94.

Comparative analysis of draft genome contiguous sequences ordered against *B. abortus* 2308W revealed a deletion due to repetitive sequences in the P1, MR, and A1 isolates representatives relative to the A2B cluster, including nine genes encoding mainly sugar transporters (BAW_20470-BAW_20476 and BAW_20479-BAW_20480) and four adjacent pseudogenes.

### Pseudogenization Is a Source of Genetic Variability That Relates to Host Preference

**Fig. 4.—:**
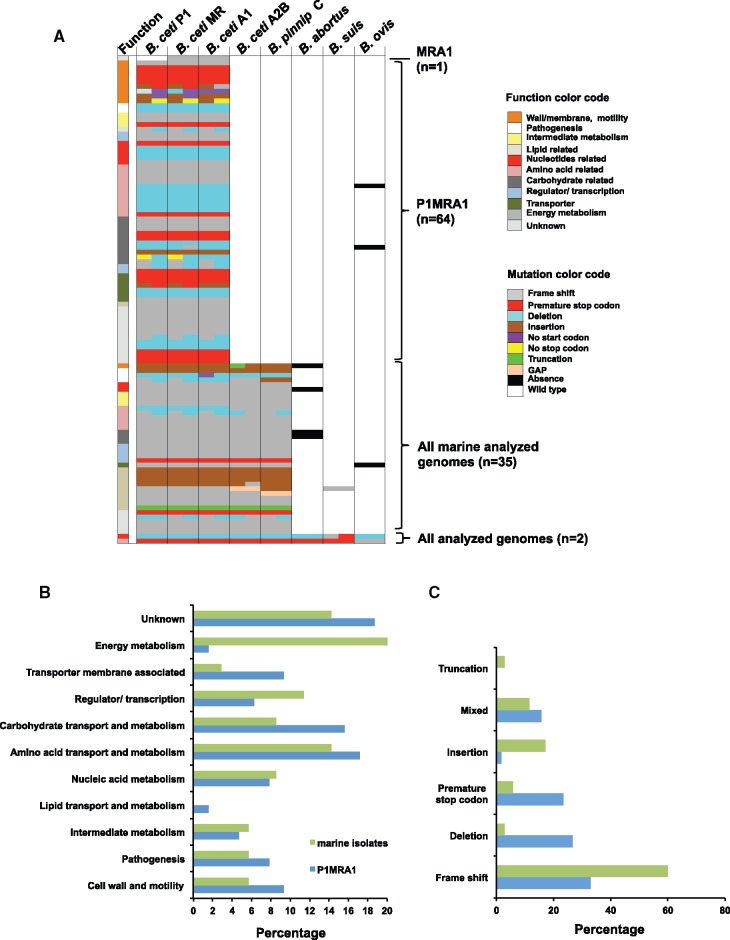
Classification of *Brucella* pseudogenes in relevant tree branching points found in representative genomes. (*A*) The left bar graph indicates function of each pseudogene according to color code and distributed according to four branches (MRA1, PMRA1, all marine analyzed genomes and all analyzed genomes). Every other bar represents the pseudogenes in each genome and colors correspond to a specific pseudogene type. “No stop codon” mutation refers to longer genes as compared with other *Brucella* reference genes. The number of pseudogenes for each branch is indicated in parenthesis. Details in Supplementary data set S4, Supplementary Material online, spreadsheet “at branch pseudo” (*B*, *C*). Proportional distribution of pseudogenes classified by their function (*B*) and by mutation type (*C*), according to two branching points (marine isolates and P1MRA1) in the phylogenetic tree.

To study correlations among pseudogene accumulation and host adaptation, we performed manual pseudogene annotation in the four *B. ceti* representative genomes, one representative *B. pinnipedialis*, *B*. *abortus*, *B. ovis*, and *B. suis* genomes and compared pseudogene traits according to genome (fig. 4*A*). In all genomes, the proportion of pseudogenes was higher in chromosome II than in the larger chromosome I (supplementary data set S4, Supplementary Material online). A total of 706 pseudogenes were found among these genomes and only two were shared among them. The mutation site within each gene was often conserved, suggesting that they occurred once in a common ancestor. The main cause of pseudogenization, was frame shift (410/706, 58%), followed by deletions (90/706, 13%) (fig. 4*A* and supplementary data set S4, Supplementary Material online). Their distribution according to former gene product, subcellular location and function is in Supplementary data set S4, Supplementary Material online.

Putative primary events targeting specific metabolic pathways that have become fixed in this population can be identified by looking at the extent of gene degradation at nodes of the phylogenetic tree (fig. 2). At the branching point between the marine mammal isolates and the *B. suis*/*B. canis*/*B. microti* clade only one shared pseudogene was found. Likewise, no shared pseudogenes were found at the branching point between the *B. ceti* A2B genotype and *B. pinnipedialis*, and only one pseudogene was shared among the *B. ceti* MR and A1 genotypes, suggesting that little gene degradation occurred when they diverged.

However, extensive pseudogenization was found among the isolates from marine mammals diverging from the *B. suis* clade (fig. 4*A*). Most of the 35 found pseudogenes, related to energy metabolism (8/35, 23%), amino acid transport and metabolism (5/35, 14%), gene regulation/transcription (4/35, 11%), or unknown function (5/35, 14%) (fig. 4*B*). Frame shift (22/35, 63%) was the main cause of pseudogenization followed by insertions (6/35, 17%) (fig. 4*C*). Functional analysis of the cognate wild type genes indicated that several pseudogenes were related to relevant metabolic pathways (Supplementary data set S4, Supplementary Material online). Notably, multiple pseudogenizations had occurred in pathways that alter fatty acid metabolism. Specifically, an acetyl-CoA acyltransferase and an acetyl-CoA C acetyltransferase very likely lost function in the marine mammal isolates. Lack of these enzymes is expected to influence fatty acids synthesis and beta-oxidation. In line with this finding, AceB, a malate synthase, catalyzing the conversion of glyoxylate to malate during the TCA, glyoxylate cycle (Zúñiga-Ripa et al. 2014) has probably lost its function. A functional glyoxylate shunt provides succinate and malate from acetyl-CoA and isocitrate for the TCA cycle and it is responsible for the bacteria ability to grow on fatty acids as carbon source (Barbier et al. 2011).

Synthesis of betaine glycine an osmoprotectant and source of carbon and nitrogen, important for *B. abortus* virulence (Lee et al. 2014) is probably affected, because two related genes lost function: Choline dehydrogenase and a glycine betaine/l-proline ABC transporter. Two more genes related to *Brucella* virulence probably lost function in the analyzed marine mammal isolates: One of the four predicted autotransporters in *Brucella* encoded by *btaE*, required for full virulence and defining a specific adhesive pole in *B. suis* (Ruiz-Ranwez et al. 2013) and the predicted sugar porin encoded by BR0833, required in *B. suis* for late stages of macrophage infection (Kohler et al. 2002).

There are 64 genes commonly pseudogenized in *B. ceti* genotypes P1, MR, and A1 representatives infecting dolphins, relative to the remaining marine mammal *brucellae* clusters (figs. 2, 4*A* and supplementary data set S4, Supplementary Material online), most of them related to amino acid transport and metabolism (11/64, 17%), carbohydrate transport and metabolism (10/64, 16%) or unknown function (12/64, 19%; fig. 4*B*). Although frame shift was still the most important mechanism of pseudogenization in this group (25/64, 39%), deletion and premature stop codons were found in higher proportions (28 and 27%, respectively) as compared with the group of all marine isolates (18 and 17%, respectively). Insertions on the other hand were not as common (2%) as in the second group (17%; fig. 4*C*).

The P1, MR, and A1 genotypes show a higher proportion of gene degradation in functions related to carbohydrate transport and metabolism as well as those encoding transporters and cell envelope biogenesis functions as compared with the shared pseudogenes in the marine mammal representatives (fig. 4*B*). Several pseudogenes were tracked to specific pathways. Neither degradation of amino acids such as cysteine, glutamine, arginine, histidine, alanine, and aspartate nor pyruvate fermentation seem essential for survival in their dolphin host. The highly conserved sigma-54 factor *rpoN*, related to control of nitrogen metabolism, shows a frame shift mutation that very likely impairs its function (Ronneau et al. 2014).

Some genes related to virulence showed mutations. Degradation of outer membrane protein encoding genes as well as the flagellum operon, was also observed in the P1MRA1 *B. ceti* as in terrestrial *Brucella* (Martín-Martín et al. 2009; Moreno and Moriyón 2006). One of the type IV secretion system VirB effectors encoding gene, *vceC* contains an internal in frame deletion, resulting in loss of 10 amino acid residues as compared with *B. abortus* 2308 VceC. This mutation was present in all 30 *B. ceti* P1MRA1 genomes studied (Supplementary data set S3, Supplementary Material online) and is different from a previous reported one in terrestrial isolates (de Jong et al. 2008). This indicates that either that particular deletion does not affect protein function or that VceC is not needed for survival in the dolphin host.

Gene *galE-1* encoding an UDP-galactose 4-epimerase related to smooth LPS biosynthesis and attenuation (Rajashekara et al. 2006) has an internal stop codon that probably renders inactive its product and could be related to the fact that some *B. ceti* isolates may appear as a “rough” phenotype (Guzmán-Verri et al. 2012). The premature stop codon was consistently found in all 30 P1MRA1 analyzed genomes.

It seems then, that when *Brucella* infects marine mammals, several important pathways related to energy, transport of metabolites and regulation/transcription are being degraded mainly via frame shift mutations. Marine isolates infecting particularly dolphin hosts showed further degradation of metabolites transport pathways as well as pathways related to cell wall/membrane/envelope biogenesis and motility, via not only frame shift mutations but also by premature stop codons and even gene absence. Altogether these findings indicate that degradation of metabolic pathways in *Brucella* is related to host preference with pseudogenization being a source of genetic variability. This is important for the establishment of host–bacterial interactions among the different *Brucella* species and their preferred hosts.

At least three barriers to successful bacterial replication and transmission exist for an intracellular pathogen in a given host population. The first is the immune system that will select for variants capable of withstanding host defenses. The second one is the intracellular milieu, which imposes conditions such as requirements for lysosome evasion, intracellular trafficking, and metabolic requirements. The third one relates to the mechanisms for transmission to other hosts, which may vary among different animal species. In the case of *Brucella* organisms from terrestrial domesticated mammals, at least two additional anthropogenic conditions may play a relevant role in biasing *brucellae* recovered from these populations: Domestication of a finite genetic line of the host species and population management controls such as vaccination and slaughter strategies (Moreno 2014). It is feasible that selection towards increased virulence, transmissibility, replication and zoonotic potential observed in *B. abortus*, *B. melitensis*, and *B. suis* (biotype 1 and 3) from domesticated animals, has taken place through successive infections in confined domesticated hosts, as proposed for the evolution of other infectious diseases (Ewald 2004).

## Conclusion

Genetic variation is evident in *Brucella* from marine mammals and manifests in a variety of ways: 1) specific IS*711* insertion patterns across the genome, 2) higher numbers of IS*711* elements compared with *Brucella* from terrestrial mammals, 3) specific SNP signatures across phylogenetic clusters, and 4) pseudogenization of metabolic pathways. These traits correlate with host preference and, in the case of *B. ceti*, with oceanic origin.

We conclude that genome decay occurs through insertion sequence element proliferation and pseudogene formation. The extensive pseudogenization found suggests that these *Brucella* isolates from wildlife are less likely to be zoonotic. Moreover, the mechanism of pseudogenization varies according to host preference. At the same time, this gene loss is a source of genetic variation within the marine isolates and results in a signature of host-association. The impact of this phenomenon in gene content variation has been described as similar to that exerted by horizontal gene transfer in nonclonal species (Bolotin and Hershberg 2015).

How humans are intervening with this process by domestication of animals is an interesting question that is not only relevant in terms of natural history of bacterial diseases but also in terms of preventive measures such as vaccination.

## Supplementary Material

Supplementary data are available at *Genome Biology and Evolution* online.

## Supplementary Material

Supplementary Data set S1

Supplementary Data set S2

Supplementary Data set S3

Supplementary Data set S4

Supplementary FiguresS1-S6
