# Supplementary material for: Brucella Genetic Variability in Wildlife Marine Mammals Populations Relates to Host Preference and Ocean Distribution
Source: Genome Biol Evol. 2017 Jul 20;9(7):1901–12. doi: 10.1093/gbe/evx137 (PMC5554395; doi:10.1093/gbe/evx137)
Supplement: Supplementary FiguresS1-S6 [file evx137_SuppFigS1-S6.pdf]

**Supplementary Fig. S1.**

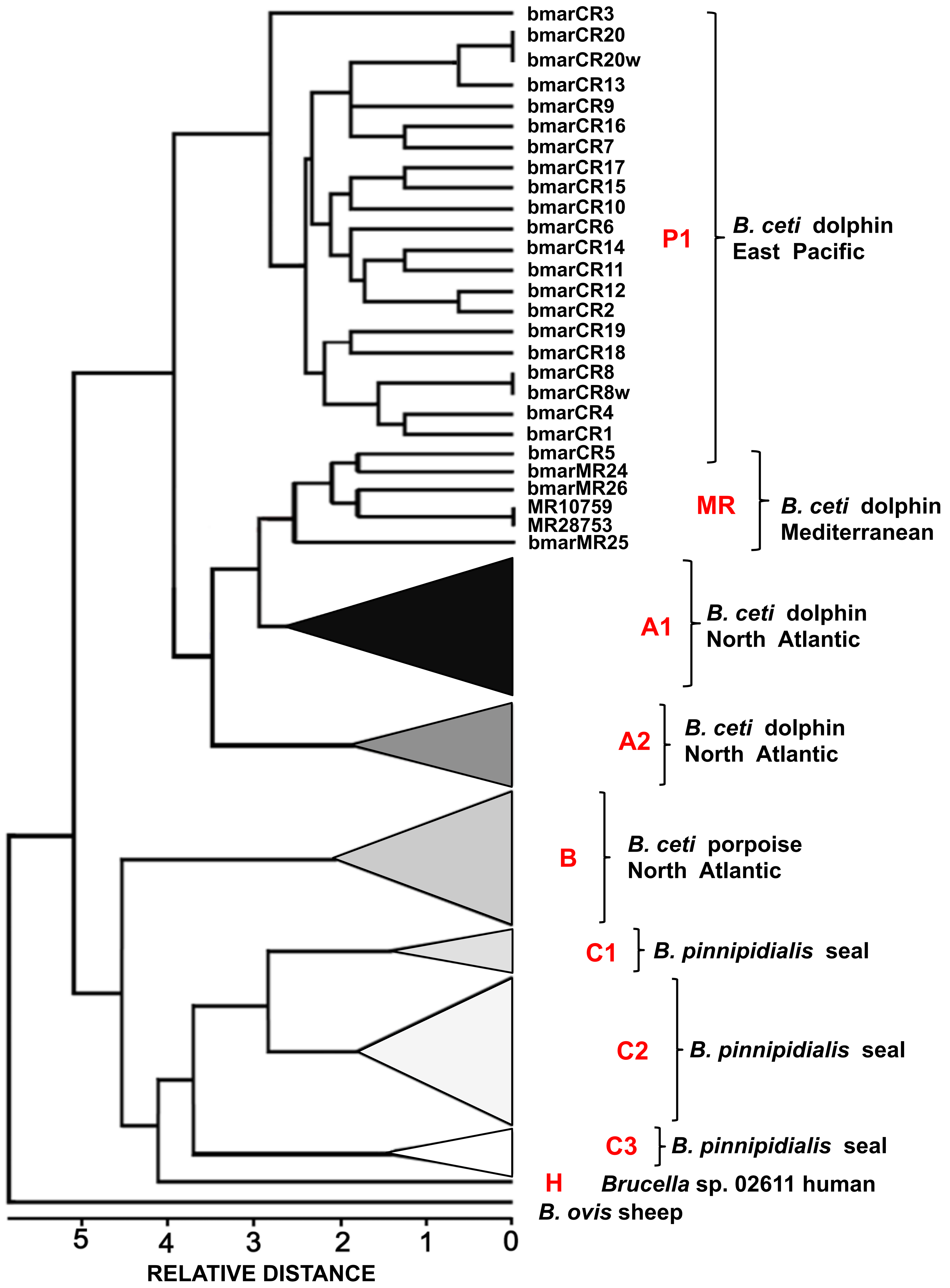


**Supplementary Fig. S1.** Cladogram based on MLVA-16 analysis of *Brucella* isolates from marine mammals constructed according to: (http://microbesgenotyping.i2bc.paris-saclay.fr/). Some clades are collapsed for clarity; for full detail, see Maquart et al., 2009). All *B. ceti* isolates from cluster P1 are from *S. coeruleoalba* dolphins inhabiting the Eastern Tropical Pacific. All *B. ceti* isolates from the Mediterranean were isolated from *S. coeruleolalba* dolphins with the exception of bmarMR25, isolated from *Tursiops truncatus* (Isidoro-Ayza et al 2014, Garofolo et al 2014). In A1, 8 out of 18 and in A2, 1 out of 8 were S. *coeruleoalba* dolphins. The *B. pinnipedialis* isolates from clusters C1 and C2 are mainly from harbor seals (*Phoca vitulina*), whereas those in cluster C3 are from hooded seals (*Cystophora cristata*, cluster C3). The H cluster is represented by a human *Brucella* sp. isolate from New Zealand (*Brucella* sp. 02611), related to other human isolates from Peru and to an isolate (*Brucella* sp. F5/99) from an aborted fetus of a bottle nose dolphin (*Tursiops truncatus* ) from San Diego, California (Whatmore et al. 2008). The *B. ovis* F8/05B was used as an out-group for the analysis.

**Supplementary Fig. S2.**

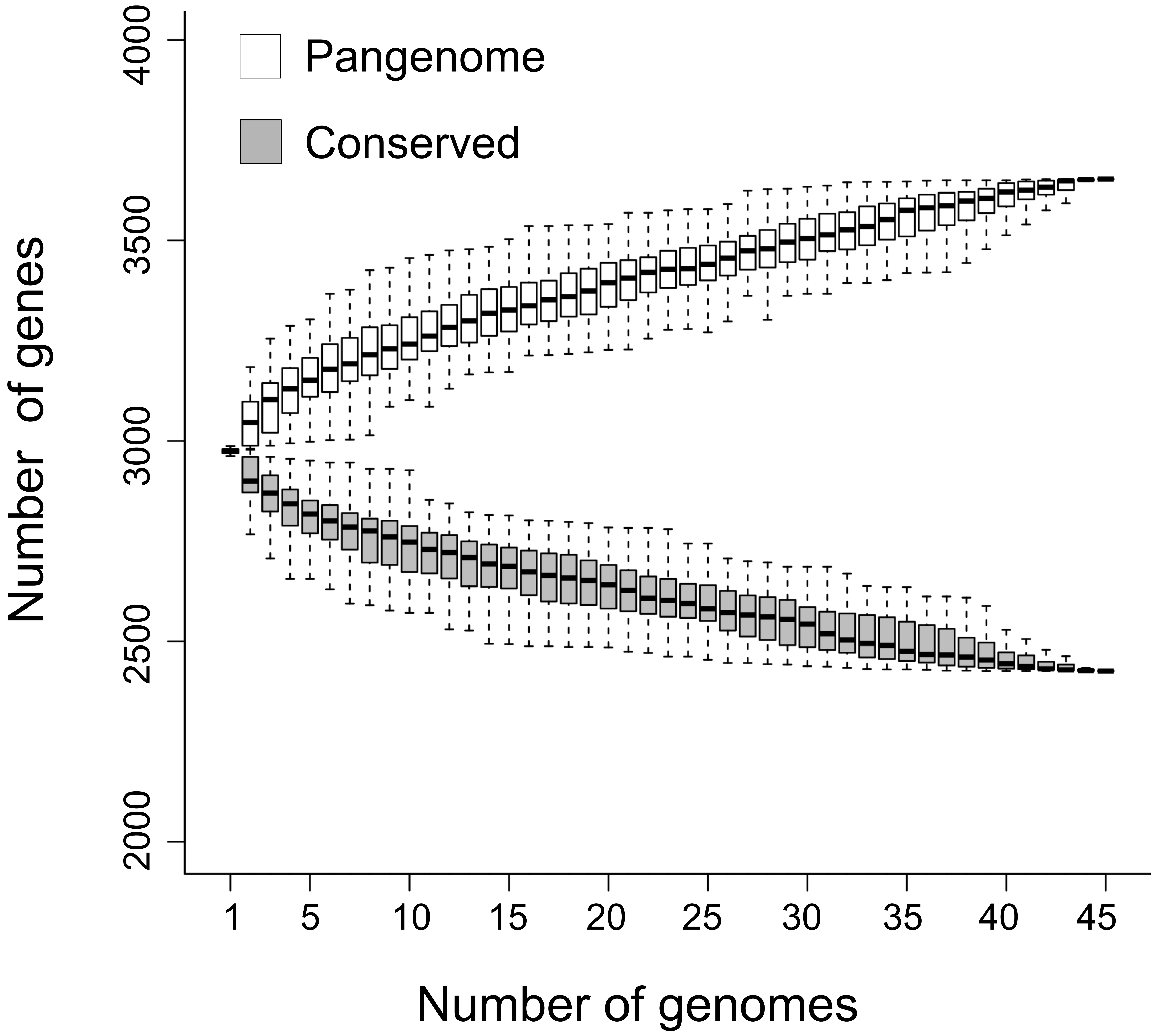


**Supplementary Fig. S2.** Number of conserved genes and genes in the pangenome. As number of included *Brucella* genomes in the analysis increased, the number of conserved and unique genes plots plateaud. This indicates that the number of genomes used in this study is representative of *Brucella* genetic content and results would not differ if more genomes included.

**Supplementary Fig. S3.**

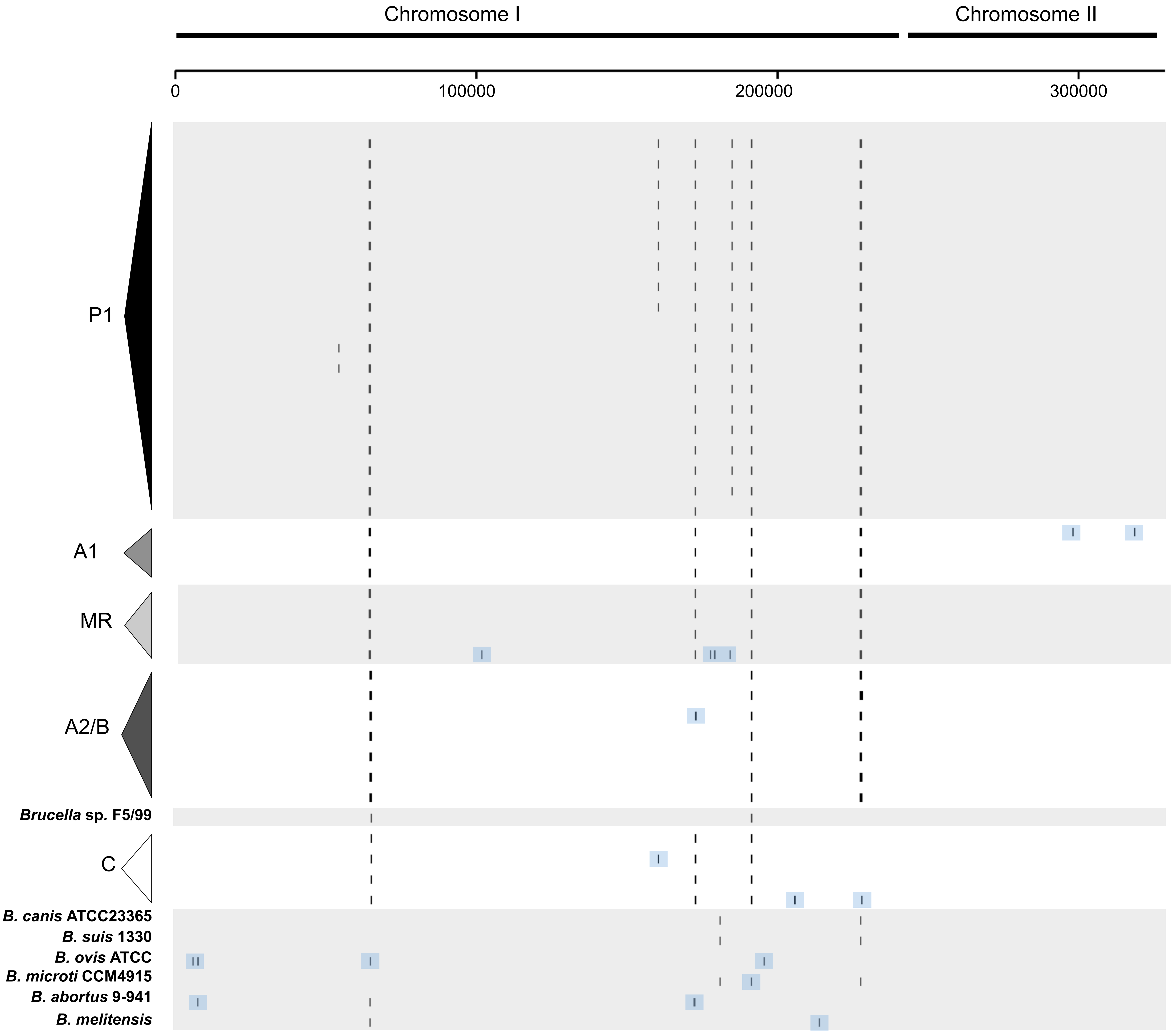


**Supplementary Fig. S3.** Recombination analysis of *Brucella* WGS throughout the length of the genome. Vertical blocks represent regions identified as predicted recombination hot spots by Gubbins and according to their position in the alignment. Approximate length in bp of the first and second chromosomes (shown as a concatenated molecule) is indicated by black bars on top of the figure, relative to *B. abortus* 9-941. The blocks shadowed in blue are unique to a single isolate, blocks are shared by multiple isolates through common descent.

**Supplementary Fig. S4.**

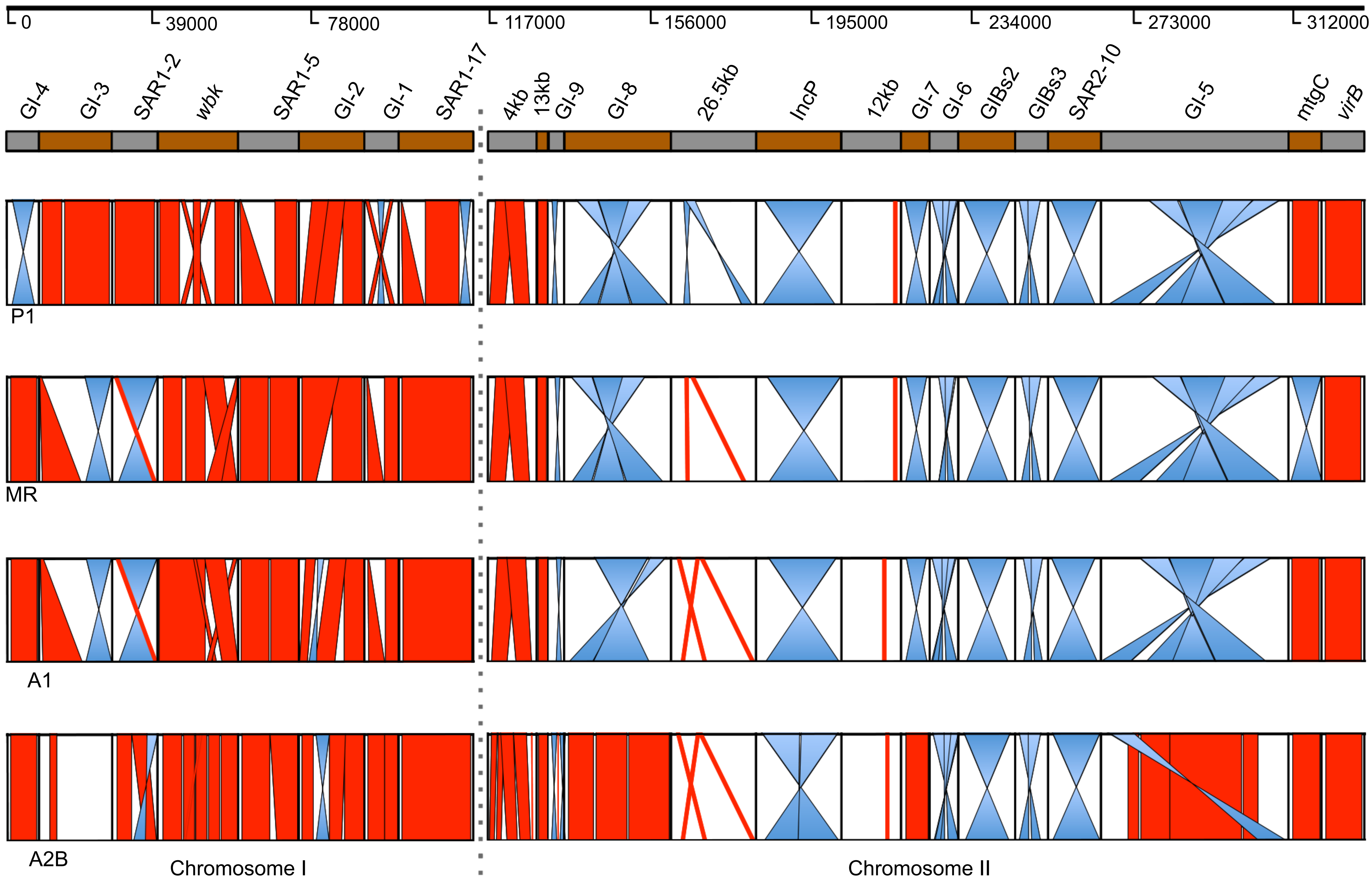


**Supplementary Fig. S4.** ACT comparison of 23 anomalous regions/genomic islands (GIs) in selected *B. ceti* genomes representing: P1: Eastern Tropical Pacific isolates from dolphins (isolate bmarCR17 from striped dolphin), MR: Mediterranean isolates from dolphins (isolate bmarMR26 from striped dolphin), A1: North Atlantic isolates mainly from dolphins (isolate M644/93/1 from common dolphin) and A2B: North Atlantic isolates from different marine mammals (isolate bmarM187/00/1 from white sided dolphin). Islands were concatenated and ordered in a pseudo-molecule according to their presence in *B. melitensis* 16M; this is represented as the upper row of alternating gray and brown blocks. Two islands absent in *B. melitensis* 16M (IncP and 26.5 kb) were ordered according to their presence in bmarCR17. Top coordinates show relative size in bp of each island. Dotted gray line represents the division of both chromosomes. Bottom lines of each comparison box show the islands' distribution in the query genomes. Abscence of segments of the islands are shown as empty spaces in the boxes. Regions present in the same position and order in the genomes (when compared with the pseudomolecule) are shown in red color, and inversions in blue. The previously reported 67kb GI specific for *B. pinnipedialis* (Maquart M et al, 2008) was not present in the shown genomes but it was found in *B. ceti* bmarMR24. GI arrangement comparison among genotypes in a genome context indicates that the MR cluster is very similar to A1, and similar to P1. The most dissimilar cluster is A2/B, with discrete differences. The GIs in chromosome II of cluster A2/B are similar to the reference sequences and at the same time is the most different when compared to the other genotypes. The original GI sequences were taken from several reference strains. *GI-4*: BMEI1919-BMEI1923; *GI-3:* BMEI1674-BMEI1703; *SAR1-2*: BMEI1656-BMEI1664; *wbk*: BMEI1393-BMEI1422; *SAR1-5*: BMEI1218-BMEI1231; *GI-2*: BMEI0993-BMEI1012; *GI-1:* BMEI0801-BMEI0907; *SAR1-17*: BMEI0051-BMEI0065; *4kb*: BruAb2_0165-BruAb2_0171; *13kb*: BruAb2_1085-BruAb2_1086; *GI-9*: BMEII0875-BMEII0878; *GI-8*: BMEII-0826-BMEII0850; *26.5kb*: BOV_A0483-BOVA-0515; *IncP*: BRA0361-BRA0380; *12kb*: BMI_II1048-BMI_II1054; *GI-7*: BMEII0811-BMEII0815; *GI-6*: BMEII0710-BMEII0719; *GIBs2*: BR0584-BR0593; *GIBs3*: BRA0630-BRA0635; *SAR2-10*: BMEII0447-BMEII0457; *GI-5*: BMEII0185-BMEII0226; *mtgC*: BRA0036-BRA0042; *virB*: BMEII0024-BME-0034.

**Supplementary Fig. S5.**

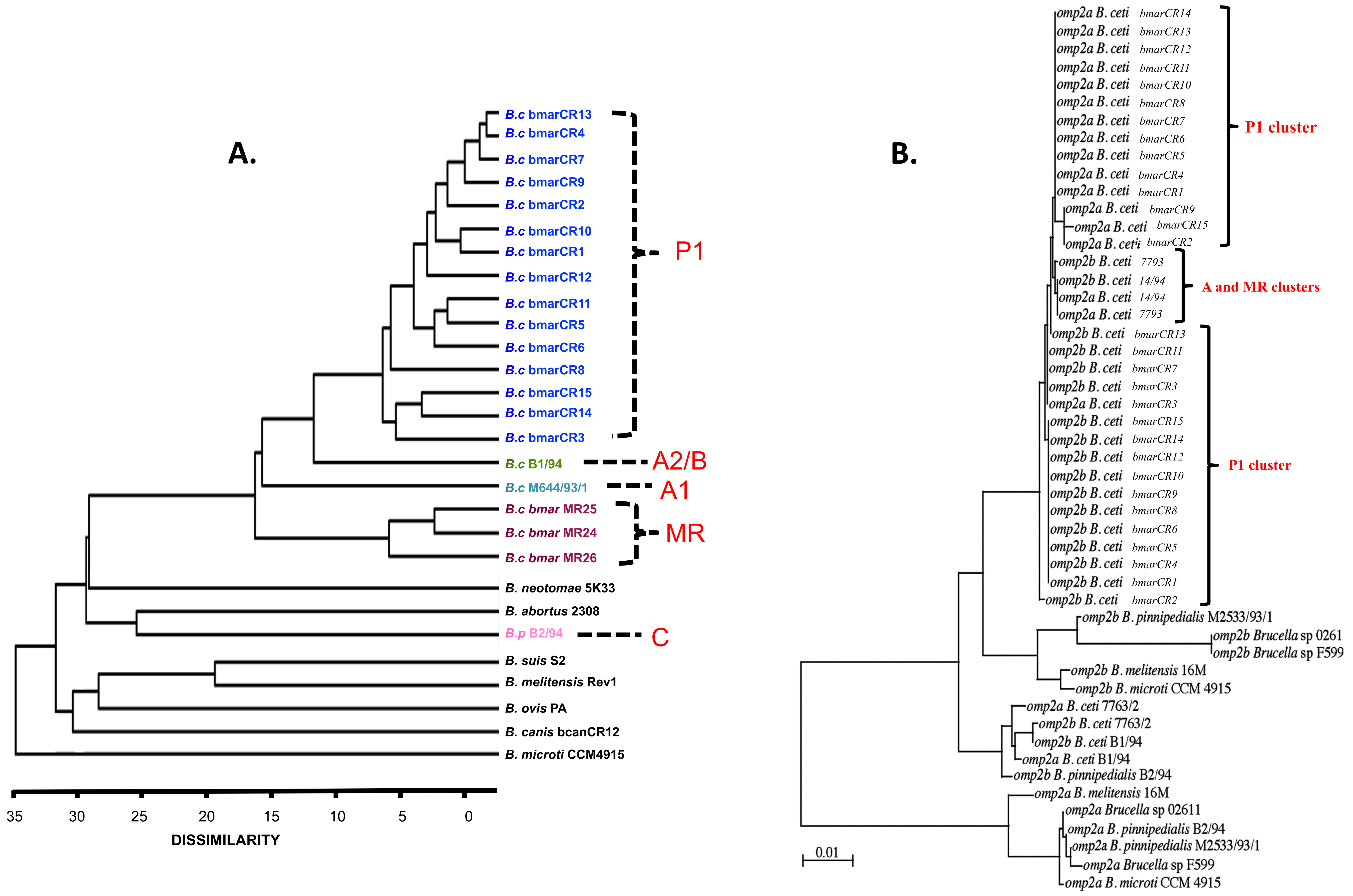


**Supplementary Fig. S5.** Cladograms derived from phenotypic analysis of *Brucella* strains. *(A)* Cladogram based on concatenated results of MALDI-TOF MS analysis and gas liquid chromatography analysis of the fatty acid methyl esters of different *Brucella* cell extracts shows clustering agreement with the phylogenetic tree in Fig. 3. Separate MALDI and TOF MS results are in Dataset S1. *(B)* Cladogram from CLUSTAL W-aligned *omp2a* and *omp2b* nucleotide sequences of *Brucella* strains. The omp2a and omp2b nucleotide sequences reveal that the Eastern Tropical Pacific *B. ceti* isolates from *S. coeruleoalba* dolphins (P1 cluster) are closely related and nearby the Atlantic/ Mediterranean *B. ceti* isolates (A and M clusters). The *B. pinnipedialis* cluster C is with terrestrial *Brucella* isolates.

**Supplementary Fig. S6.**

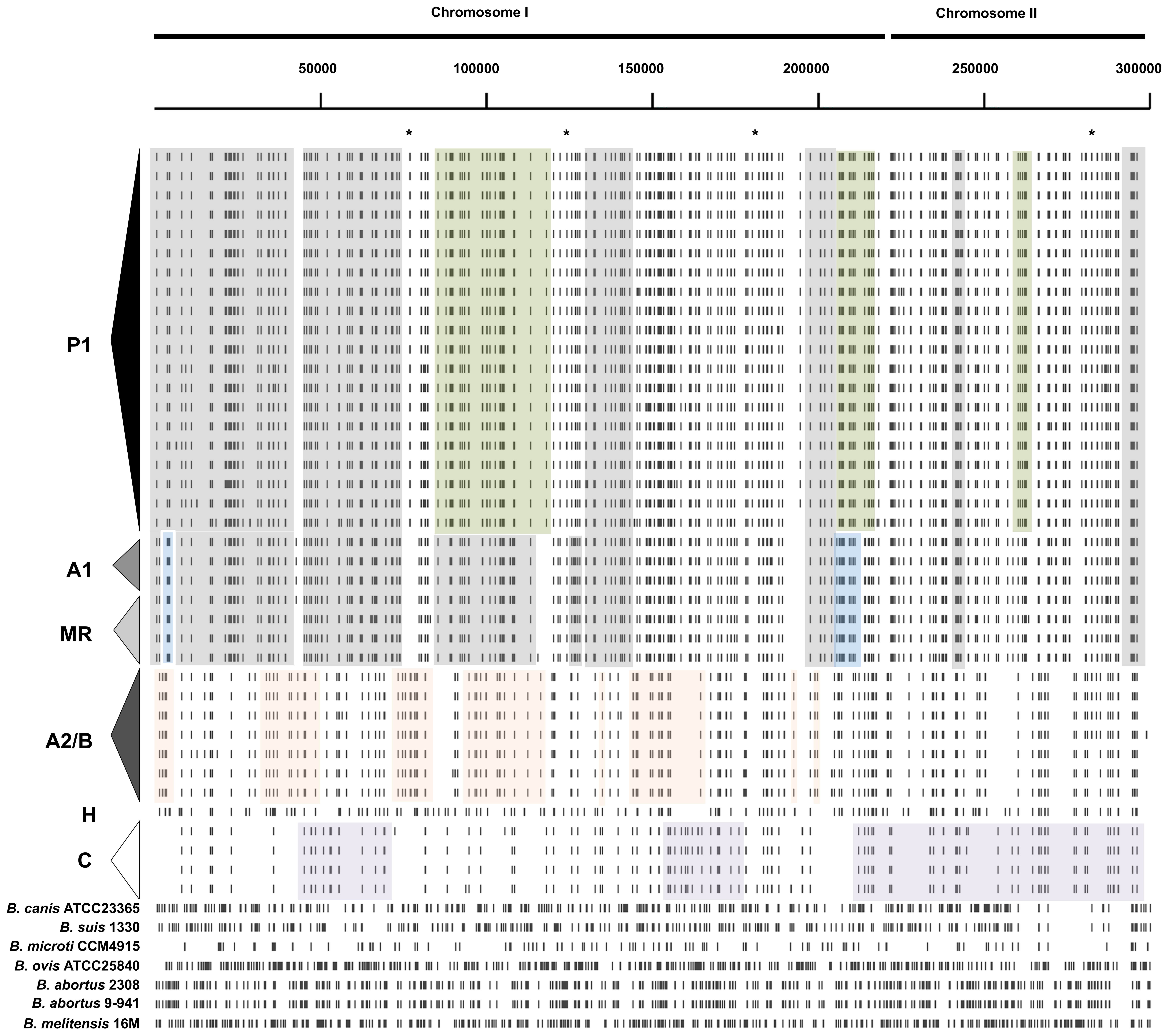


**Supplementary Fig. S6.** Reconstruction of SNPs across the phylogenetic tree relative to the shared most recent common ancestor. Approximate length in bp of the first and second chromosomes (shown as a concatenated molecule) is indicated by black bars on top of the figure, relative to *B. abortus* 9-941. For each isolate, the SNPs positions throughout the length of the genome alignment, are represented by a vertical line. The SNPs clustering pattern represented here within each genome produces a barcode that correlates with marine genotypes and geographic origin. Some particular clusters can be correlated with one or more genotypes. SNPs clusters in gray boxes correspond to P1/A1 and MR genotypes within marine isolates. Green boxes correspond to SNPs clusters specific for P1 genotype within marine isolates. Asterisk denotes SNP positions specific for P1 genotype within marine isolates. Blue boxes correspond to SNPs clusters specific for A1/MR genotype within marine isolates. Orange boxes correspond to SNPs locations specific for A2/B genotypes within marine isolates. Purple boxes correspond to SNPs clusters specific for C genotype within marine isolates. The F5/99 isolate from a bottlenose dolphin, related to human isolates is representing the H cluster.
